# Non-Coding Micro RNAs and Hypoxia-Inducible Factors Are Selenium Targets for Development of a Mechanism-Based Combination Strategy in Clear-Cell Renal Cell Carcinoma—Bench-to-Bedside Therapy

**DOI:** 10.3390/ijms19113378

**Published:** 2018-10-29

**Authors:** Youcef M. Rustum, Sreenivasulu Chintala, Farukh A. Durrani, Arup Bhattacharya

**Affiliations:** 1 Department of Internal Medicine, University of Iowa, Iowa City, IA 52242, USA; 2 Roswell Park Comprehensive Cancer Center, Department of Pharmacology & Therapeutics, Buffalo, NY 14263, USA; Arup.Bhattacharya@Roswellpark.org; 3 Neurological Surgery, Indiana University, Indianapolis, IN 46202, USA; srchinta@iu.edu; 4 Roswell Park Comprehensive Cancer Center, Department of Cell Stress Biology, Buffalo, NY 14263, USA; Farukh.Durrani@Roswellpark.org

**Keywords:** methylselenocysteine, seleno-l-methionine, clear-cell renal cell carcinoma microRNAs, hypoxia-inducible factor, antitumor activity

## Abstract

Durable response, inherent or acquired resistance, and dose-limiting toxicities continue to represent major barriers in the treatment of patients with advanced clear-cell renal cell carcinoma (ccRCC). The majority of ccRCC tumors are characterized by the loss of Von Hippel–Lindau tumor suppressor gene function, a stable expression of hypoxia-inducible factors 1α and 2α (HIFs), an altered expression of tumor-specific oncogenic microRNAs (miRNAs), a clear cytoplasm with dense lipid content, and overexpression of thymidine phosphorylase. The aim of this manuscript was to confirm that the downregulation of specific drug-resistant biomarkers deregulated in tumor cells by a defined dose and schedule of methylselenocysteine (MSC) or seleno-l-methionine (SLM) sensitizes tumor cells to mechanism-based drug combination. The inhibition of HIFs by selenium was necessary for optimal therapeutic benefit. Durable responses were achieved only when MSC was combined with sunitinib (a vascular endothelial growth factor receptor (VEGFR)-targeted biologic), topotecan (a topoisomerase 1 poison and HIF synthesis inhibitor), and S-1 (a 5-fluorouracil prodrug). The documented synergy was selenium dose- and schedule-dependent and associated with enhanced prolyl hydroxylase-dependent HIF degradation, stabilization of tumor vasculature, downregulation of 28 oncogenic miRNAs, as well as the upregulation of 12 tumor suppressor miRNAs. The preclinical results generated provided the rationale for the development of phase 1/2 clinical trials of SLM in sequential combination with axitinib in ccRCC patients refractory to standard therapies.

## 1. Introduction

Despite advances in the treatment of patients with advanced clear-cell renal cell carcinoma (ccRCC) with anti-angiogenic agents, checkpoint inhibitors, and mammalian target of rapamycin (mTOR) inhibitors alone and in combination, durable responses are seen in about 30% of treated ccRCC patients [1,2,3,4,5,6,7,8,9,10,11,12,13]. A systematic review of the first line for metastatic renal carcinoma reported an average progression-free survival of 8.4 months with a range of 6.5 to 12.3 months, and an average overall survival of 24.4 months with a range of 18.5 to 32.9 months [14]. Based on the clinical data generated in patients with advanced cancer, resistance and the associated dose-limiting toxicities remain major clinical challenges. There is an unmet clinical need to identify a new treatment modality that is patient-centric, selective, and efficacious for metastatic ccRCC patients. Both primary and metastatic ccRCC tumors are uniquely characterized by the expression of altered biomarkers associated with increased angiogenesis, metastasis, and drug resistance, including deletion and/or mutation of the von Hippel–Lindau (VHL) tumor suppressor gene in the majority of ccRCC tumors, resulting in the stable expression of hypoxia-inducible factors 1α and 2α (HIFs), and vascular endothelial growth factor (VEGF) [15,16,17,18,19,20,21,22,23,24,25,26,27,28,29,30,31,32,33]. Programmed death 1 (PD-1) is expressed in the membrane and cytoplasm of activated T cells, B cells, and dendritic cells. Programmed death ligand 1 (PD-L1) is expressed in 21–75% of ccRCC tumors, and allows cancer cells to evade immune response [34,35,36,37,38,39,40,41,42,43,44,45,46,47]. Although multiple signaling and epigenetic pathways regulate the expression of PD-L1, interferons γ and α (INF-γ and INF-α) and specific oncogenic micro RNAs (miRNAs) are also known to induce PD-L1 [48,49,50,51,52,53]. PD-L1 incidence and intensity vary among different tumor types. The analysis of melanoma tumors revealed that 38% of them express both PD-L1 and tumor-infiltrating lymphocyte (TIL), while 41% are negative for both, and 1% are PD-L1-positive, and 20% are TIL-positive [38,54,55]. PD-L1 was expressed in 69 out of 98 (70.9%) ccRCC tumors expressing mutant VHL. In all wild-type VHL tumors, 11.2% express PD-L1 [16]. HIFs and PD-L1 are co-expressed in cancer cells. Under hypoxic conditions, HIFs regulate the expression of PD-L1 by binding to the hypoxia response element in the PD-L1 proximal promoter to activate its transcription [47,48,56]. PD-L1 expression in cancer cells may, therefore, be regulated transcriptionally by HIF and post-transcriptionally by miRNAs. It is likely that the effective downregulation of HIFs would lead to the downregulation of PD-L1, resulting in an increased tumor response to subsequent treatment with anti-PD-1/PD-L1 therapies.

Thymidine phosphorylase (TP), an angiogenic protein and an enzyme required for the activation of several 5-fluorouracil (FU) prodrugs, is overexpressed by approximately 30–40% of cancers [57,58,59,60,61,62,63]. TP may function as an independent prognostic factor for increased tumor vascularity, and a target for the activation of 5-FU prodrugs. Utilizing TP to activate 5-FU prodrugs may also reduce its angiogenic activity, and may synergize with VEGF-targeting drugs. The reported overexpression of TP in ccRCC provided the opportunity to evaluate 5-FU prodrugs, such as S-1, in combination with tyrosine kinase inhibitors (TKIs) targeting VEGF/VEGF receptor (VEGFR).

Morphologically, ccRCC tumors are characterized by extensive lipid accumulation. Hypoxia-inducible protein 2 (HIG-2) is highly expressed in tumors expressing HIF1α, but not HIF2α [22,64]. Results generated indicate that HIG-2 is a direct target of HIF1α, but not HIF2α**.** Carnitine palmitoyltransferase (CPT1A), a fatty-acid transporter in the mitochondria, was recently reported to be a direct target of HIFs [65]. Clear-cell RCC cells transfected with VHL led to the downregulation of CPT1A, resulting in fatty-acid transport into the mitochondria, and forcing the formation of lipid droplets from fatty acids. Recent published reports indicated that ccRCC tumor cells expressing mutant VHL and the stable expression of HIFs participate in lipid deposition. However, HIF2α, but not HIF1α, controls the expression of perilipin 2, resulting in lipid storage [66]. In cells with a co-expression of HIFs, miRNA-155, and miRNA-210, it is possible that HIG-2, CPT1A, and perilipin 2 may also be regulated by miRNAs through HIF-dependent or -independent pathways. Since both HIFs are involved in the regulation of lipid droplets in ccRCC, agents that target HIF2α, but not HIF1α, may express limited antitumor activity. Agents that target both HIFs may have greater therapeutic impact and could avoid the need to regulate or target individual pathways regulated by HIFs.

## 2. Results

### 2.1. Hypoxia-Inducible Factors 1α and 2α (HIFs) and VHL Tumor Suppressor Gene

**Figure 1:**
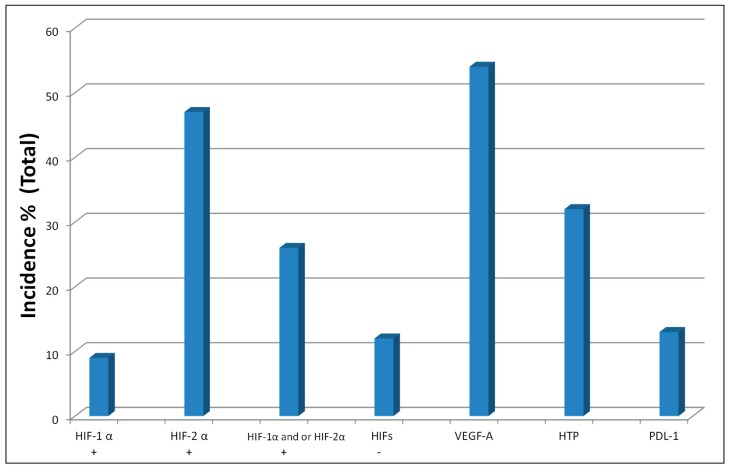
Hypoxia-inducible factors (HIFs, ‘+’ indicates presence and ‘−’ means absence), vascular endothelial growth factor (VEGF), high-intensity thymidine phosphorylase (HTP), and programmed death ligand 1 (PD-L1) biomarker expression in clear-cell renal cell carcinoma (ccRCC) tumors. The data for HIFs and VEGF were generated by our laboratory [20,22], while others are from published reports [13,63].

The molecular profiles of ccRCC tumors are summarized in Figure 1 and Table 1. HIFs are transcriptional factors that regulate the expression of over 200 genes involved in angiogenesis, tumor metastasis, and drug resistance. Unlike colorectal and head-and-neck tumors, ccRCC tumors feature a high incidence and intensity of constitutively expressed HIFs, as well as lower levels of VEGF and prolyl hydroxylase 2 (PHD2), with no detectable prolyl hydroxylase 3 (PHD3), as assessed by immunohistochemistry (Table 1).

**Figure 2:**
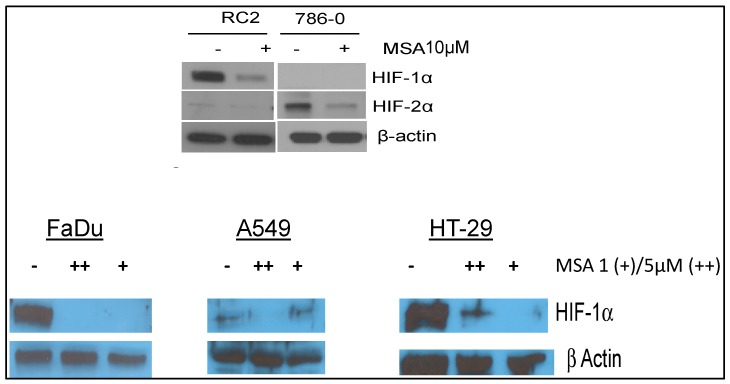
Constitutively expressed HIFs are selenium targets [20,32]. Effects of methylseleninic acid (MSA), the active moiety of methylselenocysteine (MSC) or seleno-l-methionine (SLM), on the expression levels of constitutively expressed HIFs in RC2 and 786.0 renal cell carcinoma, and on HIF1α head and neck in FaDU, lung carcinoma, A549, and colorectal carcinoma cell lines. RC2 and 786.0 cells (adopted from Reference [20]) were exposed to 10 μM MSA for 24 h, while other cells—FaDu (adopted from Reference [32]), A549, and HT29—were exposed to 0.5% O_2_ for 24 h and treated with different MSA concentrations. Cells were lysed rapidly on ice and analyzed for HIF expression by Western blot [20,32].

Our laboratory was the first to report that constitutively expressed HIF1α and HIF2α (Table 1, Figure 2) are selenium targets (adopted from References [20,32]). The data in Figure 2 show that the inhibition of constitutively expressed HIF1α and HIF2α in RC2 and 786.0 Clear-cell RCC cells, and HIF1α in FaDu head and neck [32], A548 lung carcinoma cells, and HT29 colorectal tumor cells is selenium dose-dependent and independent of the disease site/cell type. Unlike other HIF-targeting agents, selenium inhibits HIF expression via PHD-dependent degradation [20,32].

### 2.2. Tumor Vasculature

**Figure 3:**
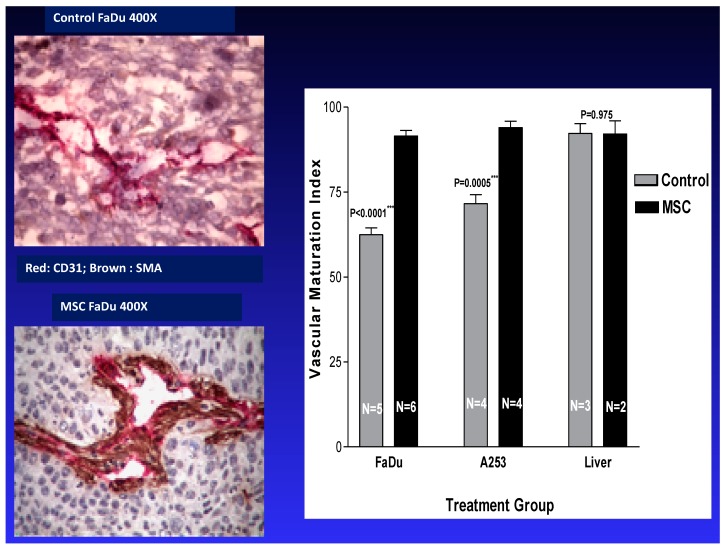
MSC selectively stabilizes tumor vasculature [68,70]. Effects of MSC treatment on the stabilization of tumor vasculature. Xenografts bearing FaDU tumors were treated orally with 10 mg/kg MSC daily for seven days, at which point the vascular maturation in tumor and normal liver tissues was assessed histologically [68,70].

To accommodate survival, growth, and metastasis, tumor cells promote the formation and development of new blood vessels [36,39]. Tumor-associated blood vessels within the tumor microenvironment are unstable and leaky, and they could represent a barrier to the delivery of effective therapies to tumor cells [67,68]. Thus, for the development of efficacious therapy, treatment should include drugs targeting biomarkers that induce the normalization of tumor-associated vasculature. Our laboratory was the first to report that the stabilization of tumor vasculature by MSC is dose- and schedule-dependent. We previously reported that the therapeutic dose and schedule of MSC/SLM exert dual effects. Firstly, anti-angiogenic effects were achieved via the inhibition of new vessel formation and a reduction in microvessel density. Secondly, tumor vascular maturation was achieved through an increase in pericyte recruitment. Collectively, these effects were associated with an increase in drug delivery and distribution to tumor cells. As shown in Figure 3, in vivo treatment with therapeutic doses of MSC resulted in a selective increase in vascular maturation index in tumors, but not in normal liver mouse tissues. The data generated demonstrate that tumor cells and their associated vasculature can be successfully and selectively modulated in vivo by a therapeutic, non-toxic dose and schedule of MSC. These results are consistent with the data generated by Jain et al., demonstrating normalization of the tumor microenvironment by Avastin, an anti-angiogenic agent [69].

### 2.3. Oncogenic miRNA-155 and miRNA-210

Non-coding miRNAs are small molecules involved in the post-transcriptional regulation of genes, and are often associated with increased angiogenesis and drug resistance. Micro RNAs function as either tumor suppressors or promoters, and they act by targeting the 3’ untranslated region (3’-UTR) of targeted genes [71,72]. Micro RNAs reduce the gene expression of mRNAs by inhibiting translation or via degradation of the transcript. Oncogenic miRNA-155 and miRNA-210 are highly overexpressed in ccRCC tumors expressing HIF1α, HIF2α, VEGF, and PD-L1 [73,74,75,76,77,78,79,80,81].

**Figure 4:**
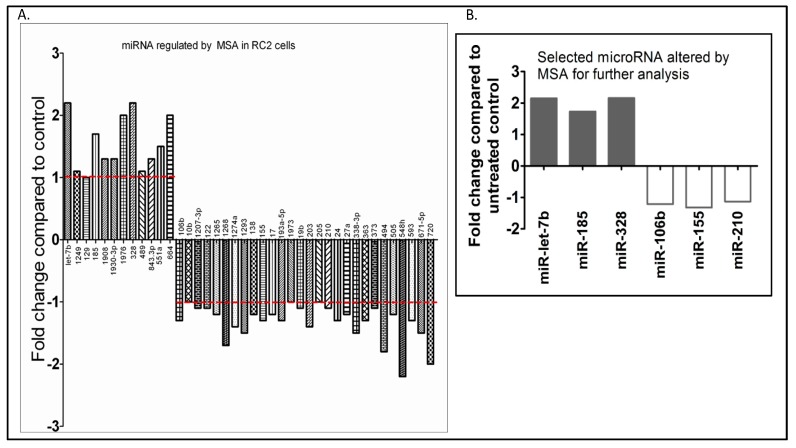
Oncogenic and tumor-suppressor micro RNAs (miRNAs) expressed in ccRCC are selenium targets. Effects of MSA on the expression levels of oncogenic and tumor-suppressor miRNAs altered in RC2 cells expressing HIF1α: (**A**) microarray analysis of miRNAs regulated by the treatment of RC2 cells with 10.0 μM MSA for 24 h, and (**B**) selected miRNAs shortlisted for further analysis.

To identify a possible link between HIF-α protein expression levels and tumor-associated miRNAs, three primary ccRCC biopsies and two ccRCC cell lines expressing a similar incidence and distribution of HIF-α were analyzed using a microarray for miRNA expression. Microarray analysis using an Exiqon microarray chip of RC2 cells treated with methylselenic acid (MSA), an inhibitor of HIF1α, revealed that 28 miRNAs were downregulated and 12 miRNAs were upregulated (Figure 4A). Although several miRNAs were altered, selected miRNAs which were upregulated and downregulated by MSA treatment are shown in Figure 4B. These results suggest that these miRNAs are likely regulated by HIF1α and can be effectively modulated by therapeutic doses of selenium.

**Figure 5:**
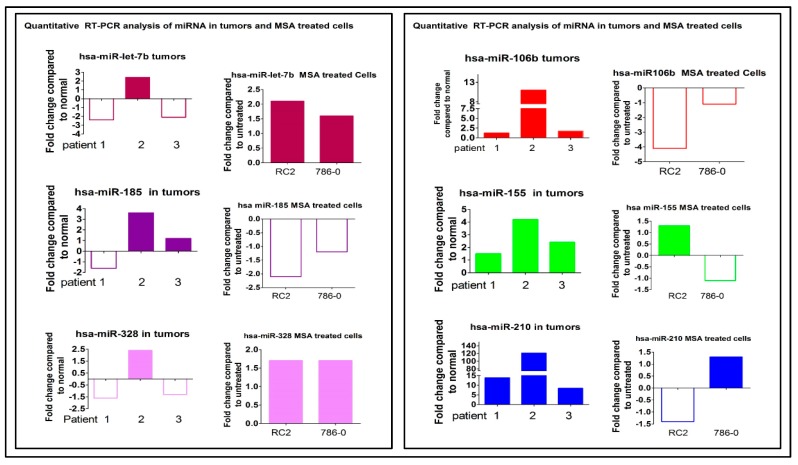
Selected miRNAs expressed in primary ccRCC biopsies are also expressed in ccRCC cell lines and can be modulated by selenium. Modulation of miRNAs expressed in ccRCC patient tumor biopsies, and in RC2 and 786.0 cells lines treated with MSA**.** Quantitative RT-PCR analysis of selective hypoxia-regulated microRNA in human RCC patient tumors (*n* = 3), and in RC2 and 786.0 cells treated with MSA. MicroRNAs downregulated in human tumors (miR let7b and miR328) (**left panel**) found to be upregulated with MSA treatment in RC2 and 786.0 cells. MicroRNAs which were upregulated (**right panel**: miR106b, miR155, and miR210; **left panel**: miR185) in RCC patients were found to be downregulated with MSA treatment in RC2 and 786.0 cells. Log fold changes are shown compared to matched normal kidney tissues for patients and untreated RC2 and 786.0 cells.

The data in Figure 5 indicate that the miRNAs that were significantly altered by MSA treatment of RC2 cells expressing HIF1α and of 786.0 cells expressing HIF2α were also altered in primary ccRCC biopsies.

Two miRNAs, Let-7b, and -328, which were upregulated, and miRNA-106b, -155, and -210, which were downregulated by MSA treatment of RC2 and 786.0 cells, were randomly selected to perform qRT-PCR analysis along with four primary ccRCC tumor biopsies and their paired normal kidney cells.

The results presented in Figure 5 confirmed the microarray data that these selected miRNAs which were altered in RC2 and 786.0 cells were similarly altered in the patient biopsies, and their expressions could be modulated in vitro and in vivo by selenium. Collectively, the data generated demonstrate that a defined dose and schedule of selenium can effectively modulate the expression levels of specific oncogenic and tumor-suppressor miRNAs altered in ccRCC tumor cells.

### 2.4. Selenium: A Selective Modulator of Anticancer Therapies

#### 2.4.1. Nude Mice Bearing HIF1α

**Figure 6:**
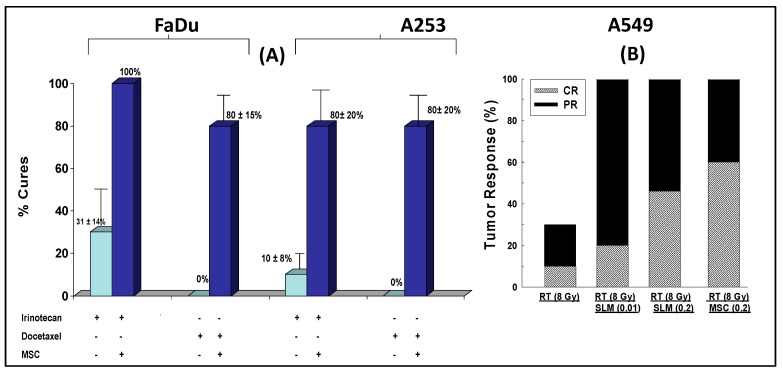
Antitumor activity of MSC in combination with irinotecan and docetaxel in nude mice bearing human head-and-neck cancer cells, FaDU and A253 (**A**), and radiation-treated A549 lung carcinoma (**B**). MSC was administered orally daily for seven days and concurrently with anticancer therapies administered on day seven [82].

The data in Figure 6A demonstrate the antitumor activity of MSC in sequential combination with two representative cytotoxic drugs, irinotecan (an approved drug for the treatment of colorectal cancer) and docetaxel (used in head-and-neck cancers among others), and radiation therapy. Oral daily administration of 10 mg/kg/day MSC for seven days prior to and concurrent with the administration of cytotoxic or radiation therapies beginning on day seven was associated with enhanced therapeutic efficacy.

The data in Figure 6B demonstrate the antitumor activity of MSC in sequential combination with radiation therapy of mice bearing A549 lung carcinoma tumors expressing HIF. Collectively, MSC was found to significantly enhance the therapeutic efficacy of chemotherapy and radiation in different human cancer xenografts from different disease sites. The results generated suggest that the action of selenium in tumor cells expressing HIFs is a universal phenomenon, irrespective of the cancer type or disease site.

#### 2.4.2. Nude Mice Bearing Tumor Xenografts That Constitutively Expressed HIF2α

**Figure 7:**
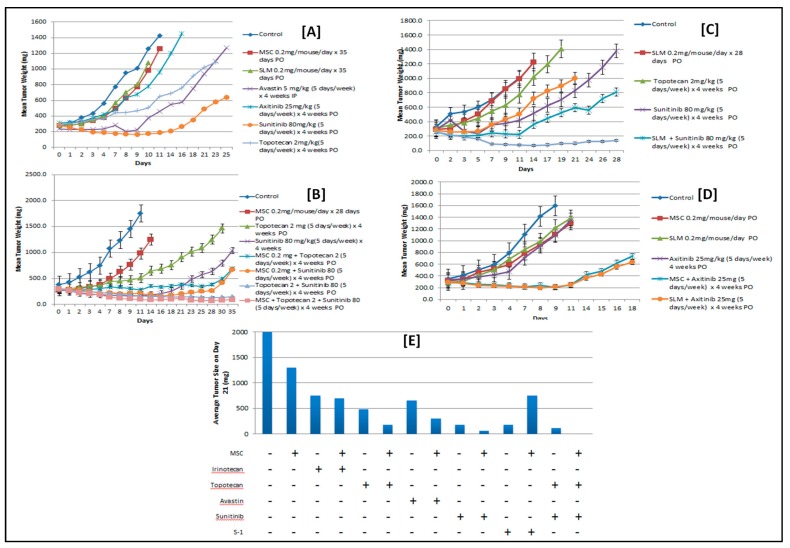
Pretreatment of 786.0 xenografts expressing HIF2α with a nontoxic but molecularly effective dose of MSC sensitizes tumor cells to subsequent treatment with the combination of VEGF/VEGF receptor (VEGFR)-targeted agents and chemotherapy. Assessment of antitumor activity of MSC, SLM, Avastin, axitinib, sunitinib, and topotecan, administered individually to nude mice bearing 786.0 ccRCC tumors (**A**), and in combination with either MSC (**B**) or SLM (**C**), and in combination with axitinib (**D**). (**E**) Summary of the antitumor activity of MSC in sequential combination with anticancer therapies [83].

Figure 7A,B depict tumor growth inhibition by MSC, SLM, axitinib, sunitinib, and topotecan. The dose and schedule of MSC and SLM that inhibited HIF exhibited limited but similar tumor growth inhibition. Sunitinib exerted greater antitumor activity than Avastin, axitinib, and topotecan [83]. The order of antitumor activity is sunitinib > Avastin ≥ axitinib > topotecan > MSC or SLM. The data in Figure 7C depict the antitumor activity of tyrosine kinase inhibitors (TKIs) that target VEGF/VEGFR, and topotecan alone and in combination with either MSC or SLM. The combination of topotecan and sunitinib in sequential combination with MSC or SLM had the most therapeutic efficacy and achieved long-term and durable responses not observed with these drugs administered individually. The data in Figure 7D indicate that MSC and SLM similarly potentiate the antitumor activity of axitinib, a Food and Drug Administration (FDA)-approved VEGFR-targeting agent for the treatment of relapsed ccRCC patients. The data in Figure 7E confirm that HIFs are a critical therapeutic target of MSC. MSC potentiates the antitumor activity of topotecan, a topoisomerase 1 poison which targets HIF synthesis, as well as that of Avastin, axitinib, and sunitinib, which target VEGF/VEGFR. In comparison, the antitumor activity of irinotecan, a topoisomerase 1 poison with no demonstrable effects on HIF protein expression, was not potentiated by MSC. In this model, S-1 exhibited significant antitumor activity, perhaps due to overexpression of TP. Collectively, the data in Figure 7E indicate that optimal therapeutic benefit was obtained with MSC in sequential combination with topotecan and sunitinib.

## 3. Discussion

Clear-cell RCCs and their associated microenvironment express a unique molecular and morphological profile including a variety of tumor-suppressor and oncogenic miRNAs. However, miRNA-155 and miRNA210 are extensively characterized and overexpressed in multiple tumor types [75,76,77,78]. Although VHL may be regulated by multiple biomarkers expressed in tumor cells and their adjacent microenvironment, miRNA-155 and -210 emerged as key modulators of VHL function, and may offer an alternative mechanism for stable expression of HIFs in ccRCC tumors [17,77]. Loss of VHL in ccRCC tumors may mimic the upregulation of HIFs by hypoxia. In recognition of the critical role of VHL in the pathogenesis of ccRCC tumors, efforts are underway to develop anti-VHL chemical agents [84,85]. Similarly, recognizing that HIFs are upregulated by hypoxia-dependent and -independent pathways and that they are critical therapeutic targets, a number of HIF inhibitors are presently under preclinical and clinical development. A recent phase 1 clinical trial of PT2385, a synthetic small-molecule HIF2α antagonist, demonstrated clinical activity in previously treated ccRCC patients [86].

Tumor microarray analysis demonstrated that HIF1α and HIF2α are individually and jointly co-expressed in a majority of primary and metastatic ccRCC biopsies [20]. In addition, it was reported that, although HIF1α and HIF2α are structurally similar, they functionally regulate different target genes in different cell types [25]. Furthermore, under hypoxia, the expression of VEGF is regulated by HIF1α, but not by HIF2α [33]. It is possible that the inhibition of one HIF isoform may induce the activation of the other in support of tumor growth. The data to date suggest that optimal therapeutic benefit may require targeting both HIF1α and HIF2α.

HIFs and PD-L1 are co-expressed in cancer cells. Under hypoxic conditions, HIFs regulate the expression of PD-L1 by binding to the hypoxia response element in the PD-L1 proximal promoter to activate its transcription [42,47]. PD-L1 expression in cancer cells may, therefore, be regulated transcriptionally by HIF and post-transcriptionally by miRNAs. It is likely that effective downregulation of HIFs would lead to the downregulation of PD-L1, resulting in an increased tumor response to subsequent treatment with anti-PD-1/PD-L1 therapies.

Micro RNA-155 and miRNA-210, amongst others, were reported to modulate the tumor microenvironment [74,75], regulate glucose metabolism [87], and target transcription factor E2F2 in ccRCC tumor cells [88]. Neal et al. reported that the VHL/HIF axis regulates the expression of several types of miRNAs in ccRCC tumors, including miRNA155 and miRNA-210 [53]. Increasing evidence suggests that oncogenic miRNA-155 and miRNA-210 are regulators of immune response biomarkers, including forkhead box P3 (FoxP3) regulatory T cell, myeloid-derived suppressor cell (MDSC) T-cells, and immune checkpoint PD-1/PD-L1 [56,80,81,89,90]. Despite the progress made in our understanding of the biology and therapeutic potential of miRNAs, their clinical use as a prognostic and as a predictor of therapeutic outcome is yet to be determined. Efforts to develop miRNA inhibitors fall short of clinical expectations [91,92,93]. The limited clinical benefits were attributed, in part, to their limited bioavailability, instability, and dose-limiting toxicities, in addition to an inability to demonstrate in vivo modulation of expression of intended targets. Our laboratory was the first to demonstrate that specific types, doses, and a schedule of MSC in ccRCC xenograft models can selectively modulate specific types of miRNAs.

Clear-cell RCC tumors are highly vascular with clear, large cytoplasms expressing perilipin 2, hypoxia-inducible lipid-droplet protein 2, which represses fatty-acid metabolism, and is a target gene of HIF1α [22,64,65]. Molecularly, the majority of ccRCC tumors express high incidence and intensity of HIF1α, HIF2α, and oncogenic miRNA-155 and -210, which target genes involved in ccRCC tumorigenesis, including VEGF and PD-L. The tumor microenvironment associated with ccRCC is leaky and unstable, expressing the common biomarkers that regulate tumor cell growth and metastasis commonly seen in many cancers. Thus, ccRCC tumors provide the opportunity to test the hypothesis and rationale for a mechanism-based treatment combination with selenium that may offer the potential for the development of novel treatment in patients with ccRCC and other cancers with similar expression of Se targets.

Resistance and dose-limiting toxicities continue to represent major clinical challenges for both cytotoxic chemotherapy and biological targeted therapies. In general, in vivo resistance is regulated by multiple molecular and immunological biomarkers expressed in tumor cells and their surrounding microenvironment. These two tumor compartments are functionally interactive. The tumor microenvironment could promote tumor growth while impeding optimal drug delivery and the distribution of effective tumor drug concentrations. Thus, the tumor microenvironment may be considered as the gatekeeper, while tumor cells are the ultimate targets. In order to achieve durable antitumor activity, treatment should include a combination of drugs that enable targeting both the tumor microenvironment and the tumor cells.

In ccRCC, HIFs, miRNA-155, and miRNA-210 are commonly co-expressed and were reported earlier to regulate the expression of gene targets implicated in enhanced angiogenesis, tumor metastasis, and resistance. While considerable efforts are underway to develop miRNA- and HIF-based strategies, in vivo toxicity, tumor instability, and limited drug delivery in effective concentrations continue to plague efforts to have a more clinically effective outcome [93]. In addition, an increased activation of 5-FU prodrugs by TP should result in increased antitumor activity [94,95,96].

**Chart 1:**
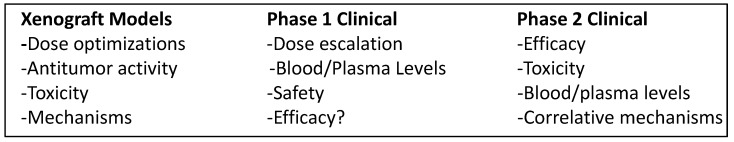
“Bench-to-bedside” therapeutic development of SLM from preclinical models to phase 1 and 2 clinical trials in patients with advanced clear-cell renal cell carcinoma (ccRCC).

During the last several years, our laboratory determined that SLM, an FDA-approved drug for clinical trials, and MSC (under development) exert several effects that are not shared by other selenium compounds and HIF-targeting compounds that are currently under preclinical and clinical evaluation [20,23,70,83,84,85,86,87,88,89,90]. We were the first to demonstrate [97,98], in several tumor xenograft models, that (1) therapeutic and nontoxic doses and a schedule of organic selenium compounds, SLM and MSC, potently enhance constitutively expressed HIF1α and HIF2α degradation; (2) SLM and MSC downregulate VEGF, which is regulated by HIF1α, but not by HIF2α; (3) SLM and MSC stabilize tumor vasculature resulting in the selective enhancement of drug delivery to tumor cells, consistent with results reported by Jain [69]; (4) SLM and MSC modulate the expression of a number of tumor-suppressor and oncogenic miRNAs altered in ccRCC tumors; (5) SLM and MSC offer selective protection against toxicity induced by toxic and often lethal doses of cytotoxic drugs in preclinical models [83]; and (6) treatment with MSC and SLM was associated with significant enhancement of the efficacy and selectivity of anticancer therapies in head-and-neck, colorectal, and renal cancer xenografts. The antitumor activity of VEFG/VEGFR-targeted therapies alone and in combination with topotecan and S-1 can be further enhanced by MSC in mice bearing VHL-deficient 786.0 ccRCC tumors expressing HIF2α, VEGF, miRNA-155, and miRNA-210. Taken together, non-toxic doses of selenium may offer the potential for the development of novel therapeutic modality. Chart 1 is an outline of the approach used in the translational development of selenium in combination with anticancer drugs in preclinical models to phase 1 and 2 clinical trials. The data generated in several xenograft models provided the rationale for the development of a phase 1 clinical trial in ccRCC patients. The aim was to confirm that the SLM dose used to yield blood selenium concentrations similar to those determined therapeutically, synergistic with anticancer drugs in the preclinical model, could be achieved clinically without toxicity. The optimal SLM dose defined in the phase 1 trial [99] was used to design a phase 2 trial of SLM in sequential combination with axitinib, aimed at assessing the efficacy and modulation of relevant molecular correlates.

**Chart 2:**
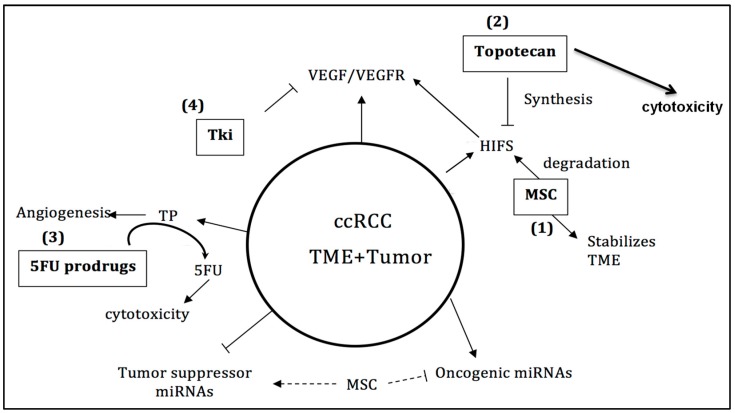
Schematic representation of targetable markers expressed in ccRCC. Methylselenocysteine (MSC) targets hypoxia-inducible factors (HIFs) and micro RNAs (miRNAs). Topotecan targets HIF synthesis, while tyrosine kinase inhibitors (TKIs) target vascular endothelial growth factor (VEGF)/VEGF receptor (VEGFR) and 5-fluorouracil (5-FU) prodrugs are the substrate for activation by thymidine phosphorylase.

Based on the preclinical results generated, a mechanism-based combination therapy is proposed, as outlined in Chart 2. In order to achieve optimal therapeutic benefit with the proposed mechanism-based drug combination, the dose, schedule, and sequence of MSC and SLM are critical parameters. Pretreatment with selenium prior to and concurrent with the administration of anticancer therapy is necessary for the optimal modulation of relevant selenium biomarkers in tumor cells and for the optimal stabilization of tumor vasculature. To maintain the optimal and sustained inhibition of HIFs and associated gene targets, it is recommended that topotecan be administered in combination with MSC or SLM. Since therapeutic doses and the schedule of selenium partially downregulate the expression levels of VEGF in tumor cells expressing HIF1α but not HIF2α [20,23], we propose, therefore, adding TKI inhibitors to the combination regimen in order for maximum downregulation of VEGF/VEGFR. This proposed mechanism-based combination was evaluated in 786.0 xenografts and was determined to be highly selective and therapeutically effective. The dose and schedule of the SLM/MSC used were selected based on their molecularly effective dose instead of the maximum tolerated dose. Furthermore, since the expression level of PD-L1 is regulated by HIFs and miRNAs, it is reasonable to expect that SLM/MSC will also modulate the therapeutic efficacy of checkpoint inhibitors. Proof of principle in ccRCC could provide the basis for the verification of this mechanism-based treatment combination in other tumors expressing these molecular targets similarly affected by SLM/MSC.

## 4. Conclusions and Future Perspectives

The aim of this paper was to determine that the levels of specific biomarkers altered in the majority of ccRCC tumors, such as HIFs, oncogenic miRNA-155 and miRNA-210, and VEGF, can be selectively downregulated by therapeutic nontoxic doses and a schedule of MSC and SLM. In addition, the aim was also to confirm that downregulation of these biomarkers would translate into therapeutic synergy with anticancer therapies. The results in several xenograft models and with multiple cytotoxic and biologic agents demonstrated that the dose- and time-dependent downregulation of constitutively expressed HIFs, miRNA-155 and -210, and VEGF-A by selenium was associated with enhanced therapeutic efficacy and selectivity of anticancer therapies. Preclinical data generated provided the rationale for the development of a phase 1 clinical trial in ccRCC patients treated with escalating doses of SLM in sequential combination with a fixed dose of axitinib [99,100]. Unlike the 200 μg/day SLM dose used in prevention clinical trials, the SLM doses used in combination therapy were 10 mg/kg in nude mice, and 8000 μg/day in ccRCC patients, which was the dose recommended for the ongoing phase 2 clinical trial for efficacy assessment and for the monitoring of the effects of SLM on relevant biomarkers. The plasma selenium concentrations achieved clinically with the recommended SLM dose were comparable with those achieved with SLM doses determined therapeutically synergistic with anticancer drugs in preclinical models. The mechanism-based drug combination proposed in Chart 2 warrants expanded preclinical investigation and clinical verification. Proof of concept that enhanced therapeutic efficacy and selectivity of axitinib in refractory ccRCC patients are SLM dose- and schedule-dependent will be highly innovative and significant. Furthermore, the ability of selenium to downregulate specific biomarkers associated with drug resistance may provide the opportunity for the clinical development of SLM in sequential combination with other clinically available targeted therapies.

## 5. Material and Methods 

### 5.1. Cell Culture and Drug Treatments

Clear-cell RCC cell lines 786.0 and RC2 were cultured in Rosewell Memorial Park Institute (RMPI-1640) medium with 10% fetal bovine serum (FBS) and 1% penicillin/streptomycin (PenStrep, Sigma-Aldrich, St. Louis, MO, USA) at 37 °C in an incubator with 5% CO_2_. Cells were routinely tested for mycoplasma contamination. Cells were seeded in T75 and/or T150 flasks, and were allowed to grow overnight. Cells were treated with MSA for 24 to 48 h, and were processed to isolate total RNA. Untreated control cells were maintained without treatment.

### 5.2. Animals

Female athymic nude mice (Envigo, nu/nu, 20–25 g body weight), 8–12 weeks of age, were used for the tumor xenograft experiment as previously described [97]. All studies were carried out as approved by the Institutional Roswell Park Comprehensive Cancer Center Animal Care and Use Committee (207M, 2009).

#### Tumor Xenografts

Clear-cell RCC 786.0 cells were cultured in RMPI-1640 and transplanted into nude mice to establish xenografts. Tumors were harvested, and ~50 mg of non-necrotic tumor tissue was transplanted into nude mice and randomized to groups of 5–10 mice each. Treatment with drugs alone or in combination was started when tumors reached ~200 mg, and the tumor volume and response were measured as described previously [97]. Drug toxicity was evaluated by measuring the weight loss of the mice biweekly.

### 5.3. Drugs

MSC and SLM (Sigma-Aldrich, St. Louis, MO, USA) were given at 0.2 mg/kg for 35 days starting seven days prior to the start of drug treatment. Axitinib (AdooQ Bioscience, Irvine, CA, USA), sunitinib (LC laboratories, Woburn, MA, USA), and topotecan (Selleckchem, Houston, TX, USA) were administered orally at 25 mg/kg, 80 mg/kg, or 2 mg/kg five days per week for four weeks, either as a single drug or in combination. Avastin (Genentech, South San Francisco, CA, USA), was given at 5 mg/kg via intraperitoneal injection for five days/week for four weeks either by itself or in combination with selenium.

### 5.4. Total RNA Isolation from ccRCC Cells Treated with and without MSA

Cells were treated with MSA for 24–48 h and processed for isolation of total RNA using Trizol reagent as per the instructions of the manufacturer (Invitrogen, Liverpool, NY, USA). RNA quantity and quality was measured using Nanodrop (Thermo-Fisher Scientific, Liverpool, NY, USA), and then used for microRNA microarray analysis and quantitative PCR analysis of microRNA.

### 5.5. Total RNA from ccRCC Patient Tumors and Their Matched Normal Tissues 

Total RNA of de-identified ccRCC patient tumors and their matched normal kidneys were obtained from the RPCI Pathology core facility. RNA samples were isolated using Trizol reagent (Thermo-Fisher Scientific, Liverpool, NY, USA) from the non-necrotic tissues selected by the pathologist, and purity was determined before use for detecting microRNA expression by qRT-PCR.

### 5.6. Reverse Transcription (RT) and miRNA qPCR

Complementary DNA (cDNA) was prepared using the following quantities of each reagent and RNA: 4 μL (20 ng) of RNA, 9 μL of H_2_O, 1 μL of Spike-In, 4 μL of reverse transcription (RT) buffer, and 2 μL of enzyme in a total volume of 20 μL. Immediately after the RT reaction was finished, a 1:80 dilution was made on the cDNA, and ROX was added. The reaction mix for qRT-PCR was prepared using 400 μL of SYBR^®^ Green Master Mix (Thermo-Fisher Scientific, Liverpool, NY, USA) and 320 μL of cDNA (from the above diluted RT reaction). Then, 9 µL of this mix was added to a 384-well plate pre-loaded with specific miR primers in triplicate using an electronic multichannel pipette. Plates were sealed with optical tape and shaken on a plate shaker for 30 s, before being centrifuged for one minute and run on the ABI7900 qPCR machine (Applied Biosystem, Foster City, CA, USA). Quantitative PCR machine cycling conditions and parameters were set exactly the same for every plate.

Normalization of Exiqon miRNA Panels (http://www.exiqon.com/mirna-pcr-panels) Excerpt from Exiqon Manual: Inter-Plate Calibrator (IPC). Since each assay was present only once on each plate, replicates were performed using separate plates. This raises the issue of run-to-run differences. To allow for simple inter-plate calibration, we designed a calibration assay with an accompanying template (annotated as UniSp3 or IPC in the plate layout files). Three wells were assigned for inter-plate calibration to provide triplicate values with the possibility for outlier removal. In each of these wells, both the primers and the DNA template were present, giving high reproducibility. The inter-plate calibrator requires only the addition of the SYBR^®^ Green master mix in order to give a signal and can, therefore, be used for quality control of each plate run.

GenEx Software (ver 6.1, Thermo-Fisher Scientific, Liverpool, NY, USA: http://www.exiqon.com/qpcr-software.

Plates were imported into the GenEx software (ver 6.1, Thermo-Fisher Scientific, Liverpool, NY, USA) and the IPCs (in triplicate on each plate) were used to normalize the plates helping to eliminate run-to-run variation when comparing multiple plates. All Ct values above 38 were set to 38 as the maximum value (this is arbitrary and may even be left blank to denoted non-amplification). All miRNAs were listed in an excel file regardless of whether or not they were expressed in the samples, with normalized Ct values for each sample. Data were represented as individual triplicate runs and as averages of triplicates (with outliers excluded). Expressions of miRNA were normalized to untreated controls, and fold changes with the selenium treatment were determined. In ccRCC patient tumors, microRNA expression was normalized to normal tissue and fold changes were determined.

## Figures and Tables

**Table 1:** Molecular profile of tumor biopsies.

**Incidence of HIF-α and PHDs Protein Expression in Primary Human ccRCC, Head & neck (H/N) and Colorectal Cancer (CRC) Tumor Biopsies:**					
**Tumors**	**HIF-1α**	**HIF-2α**	**HIF-1α and/or HIF-2α**	**PHD2**	**PHD3**
ccRCC	45% (40/88)	78% (69/88)	92% (81/88)	35% (31/88)	0% (0/88)
H/N	23% (40/173)	16% (23/146)	38% (46/122)	86% (180/210)	21% (32/153)
CRC	13% (8/62)	15% (10/65)	26% (17/64)	90% (55/61)	50% (31/62)
	**VEGF(A)**				
**Tumors**	**Incidence of Positions**	**Average Immunoscope**			
ccRCC	54% (48/88)	2.3 (weak)			
H/N	79% (136/173)	4.24 (moderate)			
CRC	97% (60/62)	5.68 (strong)			

## Abbreviations

ccRCC	Clear-cell renal cell carcinoma
HIF	Hypoxia-inducible factor
HIG-2	Hypoxia-inducible protein 2
IFN	Interferon
MSC	Se-methylselenocysteine
PD-1	Programmed death 1 receptor
PD-L1	Program death ligand 1
SLM	Seleno-l-methionine
TP	Thymidine phosphorylase
TNF	Tumor necrosis factor
TKI	Tyrosine kinase inhibitor
TIL	Tumor-infiltrating lymphocyte
VEGF	Vascular endothelial growth factor
VHL	von Hippel–Lindau

## References

[1] Albiges L., Choueiri T.K. (2017). Renal-cell carcinoma in 2016: Advances in treatment—Jostling for pole position. Nat. Rev. Clin. Oncol..

[2] Armstrong A.J., Halabi S., Eisen T., Broderick S., Stadler W.M., Jones R.J., Garcia J.A., Vaishampayan U.N., Picus J., Hawkins R.E. (2016). Everolimus versus sunitinib for patients with metastatic non-clear cell renal cell carcinoma (ASPEN): A multicentre, open-label, randomised phase 2 trial. Lancet Oncol..

[3] Escudier B., Powles T., Motzer R.J., Olencki T., Aren Frontera O., Oudard S., Rolland F., Tomczak P., Castellano D., Appleman L.J. (2018). Cabozantinib, a New Standard of Care for Patients With Advanced Renal Cell Carcinoma and Bone Metastases? Subgroup Analysis of the METEOR Trial. J. Clin. Oncol..

[4] Fernandez-Pello S., Hofmann F., Tahbaz R., Marconi L., Lam T.B., Albiges L., Bensalah K., Canfield S.E., Dabestani S., Giles R.H. (2017). A Systematic Review and Meta-analysis Comparing the Effectiveness and Adverse Effects of Different Systemic Treatments for Non-clear Cell Renal Cell Carcinoma. Eur. Urol..

[5] Greef B., Eisen T. (2016). Medical treatment of renal cancer: New horizons. Br. J. Cancer.

[6] Hsieh J.J., Chen D., Wang P.I., Marker M., Redzematovic A., Chen Y.B., Selcuklu S.D., Weinhold N., Bouvier N., Huberman K.H. (2017). Genomic Biomarkers of a Randomized Trial Comparing First-line Everolimus and Sunitinib in Patients with Metastatic Renal Cell Carcinoma. Eur. Urol..

[7] Larkin J., Hodi F.S., Wolchok J.D. (2015). Combined Nivolumab and Ipilimumab or Monotherapy in Untreated Melanoma. N. Engl. J. Med..

[8] Motzer R.J., Escudier B., McDermott D.F., George S., Hammers H.J., Srinivas S., Tykodi S.S., Sosman J.A., Procopio G., Plimack E.R. (2015). Nivolumab versus Everolimus in Advanced Renal-Cell Carcinoma. N. Engl. J. Med..

[9] Powles T., Albiges L., Staehler M., Bensalah K., Dabestani S., Giles R.H., Hofmann F., Hora M., Kuczyk M.A., Lam T.B. (2017). Updated European Association of Urology Guidelines Recommendations for the Treatment of First-line Metastatic Clear Cell Renal Cancer. Eur. Urol..

[10] Robert C., Long G.V., Brady B., Dutriaux C., Maio M., Mortier L., Hassel J.C., Rutkowski P., McNeil C., Kalinka-Warzocha E. (2015). Nivolumab in previously untreated melanoma without BRAF mutation. N. Engl. J. Med..

[11] Tannir N.M., Jonasch E., Albiges L., Altinmakas E., Ng C.S., Matin S.F., Wang X., Qiao W., Dubauskas Lim Z., Tamboli P. (2016). Everolimus Versus Sunitinib Prospective Evaluation in Metastatic Non-Clear Cell Renal Cell Carcinoma (ESPN): A Randomized Multicenter Phase 2 Trial. Eur. Urol..

[12] Xu K.Y., Wu S. (2015). Update on the treatment of metastatic clear cell and non-clear cell renal cell carcinoma. Biomark Res..

[13] Choueiri T.K., Figueroa D.J., Fay A.P., Signoretti S., Liu Y., Gagnon R., Deen K., Carpenter C., Benson P., Ho T.H. (2015). Correlation of PD-L1 tumor expression and treatment outcomes in patients with renal cell carcinoma receiving sunitinib or pazopanib: Results from COMPARZ, a randomized controlled trial. Clin. Cancer Res..

[14] Wallis C.J.D., Klaassen Z., Bhindi B., Ye X.Y., Chandrasekar T., Farrell A.M., Goldberg H., Boorjian S.A., Leibovich B., Kulkarni G.S. (2018). First-line Systemic Therapy for Metastatic Renal Cell Carcinoma: A Systematic Review and Network Meta-analysis. Eur. Urol..

[15] Kammerer-Jacquet S.F., Brunot A., Pladys A., Bouzille G., Dagher J., Medane S., Peyronnet B., Mathieu R., Verhoest G., Bensalah K. (2017). Synchronous Metastatic Clear-Cell Renal Cell Carcinoma: A Distinct Morphologic, Immunohistochemical, and Molecular Phenotype. Clin. Genitourin Cancer.

[16] Kammerer-Jacquet S.F., Crouzet L., Brunot A., Dagher J., Pladys A., Edeline J., Laguerre B., Peyronnet B., Mathieu R., Verhoest G. (2017). Independent association of PD-L1 expression with noninactivated VHL clear cell renal cell carcinoma-A finding with therapeutic potential. Int. J. Cancer.

[17] Kong W., He L., Richards E.J., Challa S., Xu C.X., Permuth-Wey J., Lancaster J.M., Coppola D., Sellers T.A., Djeu J.Y. (2014). Upregulation of miRNA-155 promotes tumour angiogenesis by targeting VHL and is associated with poor prognosis and triple-negative breast cancer. Oncogene.

[18] Nickerson M.L., Jaeger E., Shi Y., Durocher J.A., Mahurkar S., Zaridze D., Matveev V., Janout V., Kollarova H., Bencko V. (2008). Improved identification of von Hippel-Lindau gene alterations in clear cell renal tumors. Clin. Cancer Res..

[19] Melendez-Rodriguez F., Roche O., Sanchez-Prieto R., Aragones J. (2018). Hypoxia-Inducible Factor 2-Dependent Pathways Driving Von Hippel-Lindau-Deficient Renal Cancer. Front. Oncol..

[20] Chintala S., Najrana T., Toth K., Cao S., Durrani F.A., Pili R., Rustum Y.M. (2012). Prolyl hydroxylase 2 dependent and Von-Hippel-Lindau independent degradation of Hypoxia-inducible factor 1 and 2α by selenium in clear cell renal cell carcinoma leads to tumor growth inhibition. BMC Cancer.

[21] Schodel J., Grampp S., Maher E.R., Moch H., Ratcliffe P.J., Russo P., Mole D.R. (2016). Hypoxia, Hypoxia-inducible Transcription Factors, and Renal Cancer. Eur. Urol..

[22] Toth K., Chintala S., Rustum Y.M. (2014). Constitutive expression of HIF-alpha plays a major role in generation of clear-cell phenotype in human primary and metastatic renal carcinoma. Appl. Immunohistochem. Mol. Morphol..

[23] Yin M.B., Li Z.R., Toth K., Cao S., Durrani F.A., Hapke G., Bhattacharya A., Azrak R.G., Frank C., Rustum Y.M. (2006). Potentiation of irinotecan sensitivity by Se-methylselenocysteine in an in vivo tumor model is associated with downregulation of cyclooxygenase-2, inducible nitric oxide synthase, and hypoxia-inducible factor 1-alpha expression, resulting in reduced angiogenesis. Oncogene.

[24] Kondo K., Klco J., Nakamura E., Lechpammer M., Kaelin W.G. (2002). Inhibition of HIF is necessary for tumor suppression by the von Hippel-Lindau protein. Cancer Cell.

[25] Raval R.R., Lau K.W., Tran M.G., Sowter H.M., Mandriota S.J., Li J.L., Pugh C.W., Maxwell P.H., Harris A.L., Ratcliffe P.J. (2005). Contrasting properties of hypoxia-inducible factor 1 (HIF-1) and HIF-2 in von Hippel-Lindau-associated renal cell carcinoma. Mol. Cell. Boil..

[26] del Peso L., Castellanos M.C., Temes E., Martin-Puig S., Cuevas Y., Olmos G., Landazuri M.O. (2003). The von Hippel Lindau/hypoxia-inducible factor (HIF) pathway regulates the transcription of the HIF-proline hydroxylase genes in response to low oxygen. J. Boil. Chem..

[27] Gossage L., Eisen T., Maher E.R. (2015). VHL, the story of a tumour suppressor gene. Nat. Rev. Cancer.

[28] Kaelin W.G. (2002). Molecular basis of the VHL hereditary cancer syndrome. Nat. Rev. Cancer.

[29] Linehan W.M., Lerman M.I., Zbar B. (1995). Identification of the von Hippel-Lindau (VHL) gene. Its role in renal cancer. JAMA.

[30] Yao X., Tan J., Lim K.J., Koh J., Ooi W.F., Li Z., Huang D., Xing M., Chan Y.S., Qu J.Z. (2017). VHL Deficiency Drives Enhancer Activation of Oncogenes in Clear Cell Renal Cell Carcinoma. Cancer Discov..

[31] Razafinjatovo C., Bihr S., Mischo A., Vogl U., Schmidinger M., Moch H., Schraml P. (2016). Characterization of VHL missense mutations in sporadic clear cell renal cell carcinoma: Hotspots, affected binding domains, functional impact on pVHL and therapeutic relevance. BMC Cancer.

[32] Chintala S., Toth K., Cao S., Durrani F.A., Vaughan M.M., Jensen R.L., Rustum Y.M. (2010). Se-methylselenocysteine sensitizes hypoxic tumor cells to irinotecan by targeting hypoxia-inducible factor 1 alpha. Cancer Chemother. Pharmacol..

[33] Hu C.J., Wang L.Y., Chodosh L.A., Keith B., Simon M.C. (2003). Differential roles of hypoxia-inducible factor 1 alpha (HIF-1alpha) and HIF-2alpha in hypoxic gene regulation. Mol. Cell. Boil..

[34] Alsaab H.O., Sau S., Alzhrani R., Tatiparti K., Bhise K., Kashaw S.K., Iyer A.K. (2017). PD-1 and PD-L1 Checkpoint Signaling Inhibition for Cancer Immunotherapy: Mechanism, Combinations, and Clinical Outcome. Front. Pharmacol..

[35] Senthebane D.A., Rowe A., Thomford N.E., Shipanga H., Munro D., Mazeedi M., Almazyadi H.A.M., Kallmeyer K., Dandara C., Pepper M.S. (2017). The Role of Tumor Microenvironment in Chemoresistance: To Survive, Keep Your Enemies Closer. Int. J. Mol. Sci..

[36] Forster J.C., Harriss-Phillips W.M., Douglass M.J., Bezak E. (2017). A review of the development of tumor vasculature and its effects on the tumor microenvironment. Hypoxia.

[37] Noman M.Z., Chouaib S. (2014). Targeting hypoxia at the forefront of anticancer immune responses. Oncoimmunology.

[38] Baine M.K., Turcu G., Zito C.R., Adeniran A.J., Camp R.L., Chen L., Kluger H.M., Jilaveanu L.B. (2015). Characterization of tumor infiltrating lymphocytes in paired primary and metastatic renal cell carcinoma specimens. Oncotarget.

[39] Schaaf M.B., Garg A.D., Agostinis P. (2018). Defining the role of the tumor vasculature in antitumor immunity and immunotherapy. Cell Death Dis..

[40] Abbas M., Steffens S., Bellut M., Eggers H., Grosshennig A., Becker J.U., Wegener G., Schrader A.J., Grunwald V., Ivanyi P. (2016). Intratumoral expression of programmed death ligand 1 (PD-L1) in patients with clear cell renal cell carcinoma (ccRCC). Med. Oncol..

[41] Callea M., Albiges L., Gupta M., Cheng S.C., Genega E.M., Fay A.P., Song J., Carvo I., Bhatt R.S., Atkins M.B. (2015). Differential Expression of PD-L1 between Primary and Metastatic Sites in Clear-Cell Renal Cell Carcinoma. Cancer Immunol. Res..

[42] Chen J., Jiang C.C., Jin L., Zhang X.D. (2016). Regulation of PD-L1: A novel role of pro-survival signalling in cancer. Ann. Oncol..

[43] Choueiri T.K., Fay A.P., Gray K.P., Callea M., Ho T.H., Albiges L., Bellmunt J., Song J., Carvo I., Lampron M. (2014). PD-L1 expression in nonclear-cell renal cell carcinoma. Ann. Oncol..

[44] Jilaveanu L.B., Shuch B., Zito C.R., Parisi F., Barr M., Kluger Y., Chen L., Kluger H.M. (2014). PD-L1 Expression in Clear Cell Renal Cell Carcinoma: An Analysis of Nephrectomy and Sites of Metastases. J. Cancer.

[45] Joseph R.W., Millis S.Z., Carballido E.M., Bryant D., Gatalica Z., Reddy S., Bryce A.H., Vogelzang N.J., Stanton M.L., Castle E.P. (2015). PD-1 and PD-L1 Expression in Renal Cell Carcinoma with Sarcomatoid Differentiation. Cancer Immunol. Res..

[46] Leite K.R., Reis S.T., Junior J.P., Zerati M., Gomes Dde O., Camara-Lopes L.H., Srougi M. (2015). PD-L1 expression in renal cell carcinoma clear cell type is related to unfavorable prognosis. Diagn. Pathol..

[47] Messai Y., Gad S., Noman M.Z., Le Teuff G., Couve S., Janji B., Kammerer S.F., Rioux-Leclerc N., Hasmim M., Ferlicot S. (2016). Renal Cell Carcinoma Programmed Death-ligand 1, a New Direct Target of Hypoxia-inducible Factor-2 Alpha, is Regulated by von Hippel-Lindau Gene Mutation Status. Eur. Urol..

[48] Ruf M., Moch H., Schraml P. (2016). PD-L1 expression is regulated by hypoxia inducible factor in clear cell renal cell carcinoma. Int. J. Cancer.

[49] Sun C., Mezzadra R., Schumacher T.N. (2018). Regulation and Function of the PD-L1 Checkpoint. Immunity.

[50] Lastwika K.J., Wilson W., Li Q.K., Norris J., Xu H., Ghazarian S.R., Kitagawa H., Kawabata S., Taube J.M., Yao S. (2016). Control of PD-L1 Expression by Oncogenic Activation of the AKT-mTOR Pathway in Non-Small Cell Lung Cancer. Cancer Res..

[51] Mimura K., Teh J.L., Okayama H., Shiraishi K., Kua L.F., Koh V., Smoot D.T., Ashktorab H., Oike T., Suzuki Y. (2018). PD-L1 expression is mainly regulated by interferon gamma associated withJAK-STAT pathway in gastric cance. Cancer Sci..

[52] Wang X., Yang L., Huang F., Zhang Q., Liu S., Ma L., You Z. (2017). Inflammatory cytokines IL-17 and TNF-alpha up-regulate PD-L1 expression in human prostate and colon cancer cells. Immunol. Lett..

[53] Neal C.S., Michael M.Z., Rawlings L.H., Van der Hoek M.B., Gleadle J.M. (2010). The VHL-dependent regulation of microRNAs in renal cancer. BMC Med..

[54] Teng M.W., Ngiow S.F., Ribas A., Smyth M.J. (2015). Classifying Cancers Based on T-cell Infiltration and PD-L1. Cancer Res..

[55] Kaunitz G.J., Cottrell T.R., Lilo M., Muthappan V., Esandrio J., Berry S., Xu H., Ogurtsova A., Anders R.A., Fischer A.H. (2017). Melanoma subtypes demonstrate distinct PD-L1 expression profiles. Lab. Investig..

[56] Noman M.Z., Janji B., Hu S., Wu J.C., Martelli F., Bronte V., Chouaib S. (2015). Tumor-Promoting Effects of Myeloid-Derived Suppressor Cells Are Potentiated by Hypoxia-Induced Expression of miR-210. Cancer Res..

[57] Miscoria M., Di Loreto C., Puglisi F., Murray P.G., Deroma L., Atmadini M. (2012). Thymidine phosphorylase expression in metastatic kidney cancer as a potential predictor of outcome in patients treated with sunitinib. J. Clin. Oncol..

[58] Eda H., Fujimoto K., Watanabe S., Ura M., Hino A., Tanaka Y., Wada K., Ishitsuka H. (1993). Cytokines induce thymidine phosphorylase expression in tumor cells and make them more susceptible to 5′-deoxy-5-fluorouridine. Cancer Chemother. Pharmacol..

[59] Huang X., Wang L., Chen Y., Zheng X., Wang X. (2017). Poor Prognosis Associated with High Levels of Thymidine Phosphorylase and Thrombocytosis in Patients with Renal Cell Carcinoma. Urol. Int..

[60] Lin S., Lai H., Qin Y., Chen J., Lin Y. (2015). Thymidine phosphorylase and hypoxia-inducible factor 1-alpha expression in clinical stage II/III rectal cancer: Association with response to neoadjuvant chemoradiation therapy and prognosis. Int. J. Clin. Exp. Pathol..

[61] Atrih A., Mudaliar M.A.V., Zakikhani P., Lamont D.J., Huang J.T.-J., Bray S.E., Barton G., Fleming S., Nabi G. (2014). Quantitative proteomics in resected renal cancer tissue for biomarker discovery and profiling. Br. J. Cancer.

[62] Padrik P., Saar H. (2010). Thymidine phosphorylase as a prognostic factor in renal cell carcinoma. Int. Urol. Nephrol..

[63] Takayama T., Mugiya S., Sugiyama T., Aoki T., Furuse H., Liu H., Hirano Y., Kai F., Ushiyama T., Ozono S. (2006). High levels of thymidine phosphorylase as an independent prognostic factor in renal cell carcinoma. Jpn. J. Clin. Oncol..

[64] Gimm T., Wiese M., Teschemacher B., Deggerich A., Schodel J., Knaup K.X., Hackenbeck T., Hellerbrand C., Amann K., Wiesener M.S. (2010). Hypoxia-inducible protein 2 is a novel lipid droplet protein and a specific target gene of hypoxia-inducible factor-1. FASEB J..

[65] Du W., Zhang L., Brett-Morris A., Aguila B., Kerner J., Hoppel C.L., Puchowicz M., Serra D., Herrero L., Rini B.I. (2017). HIF drives lipid deposition and cancer in ccRCC via repression of fatty acid metabolism. Nat. Commun..

[66] Cao Q., Ruan H., Wang K., Song Z., Bao L., Xu T., Xiao H., Wang C., Cheng G., Tong J. (2018). Overexpression of PLIN2 is a prognostic marker and attenuate tumor progression in clear cell renal cell carcinoma. Int J. Oncol..

[67] Azrak R.G., Cao S., Durrani F.A., Toth K., Bhattacharya A., Rustum Y.M. (2011). Augmented therapeutic efficacy of irinotecan is associated with enhanced drug accumulation. Cancer Lett..

[68] Bhattacharya A., Seshadri M., Oven S.D., Toth K., Vaughan M.M., Rustum Y.M. (2008). Tumor vascular maturation and improved drug delivery induced by methylselenocysteine leads to therapeutic synergy with anticancer drugs. Clin. Cancer Res..

[69] Jain R.K. (2013). Normalizing tumor microenvironment to treat cancer: Bench to bedside to biomarkers. J. Clin. Oncol..

[70] Rustum Y.M., Toth K., Seshadri M., Sen A., Durrani F.A., Stott E., Morrison C.D., Cao S., Bhattacharya A. (2010). Architectural heterogeneity in tumors caused by differentiation alters intratumoral drug distribution and affects therapeutic synergy of antiangiogenic organoselenium compound. J. Oncol..

[71] Lytle J.R., Yario T.A., Steitz J.A. (2007). Target mRNAs are repressed as efficiently by microRNA-binding sites in the 5′ UTR as in the 3′ UTR. Proc. Natl. Acad. Sci. USA.

[72] Li Z., Rana T.M. (2014). Therapeutic targeting of microRNAs: Current status and future challenges. Nat. Rev. Drug Discov..

[73] Gulyaeva L.F., Kushlinskiy N.E. (2016). Regulatory mechanisms of microRNA expression. J. Transl. Med..

[74] Kuninty P.R., Schnittert J., Storm G., Prakash J. (2016). MicroRNA Targeting to Modulate Tumor Microenvironment. Front. Oncol..

[75] Li M., Wang Y., Song Y., Bu R., Yin B., Fei X., Guo Q., Wu B. (2015). MicroRNAs in renal cell carcinoma: A systematic review of clinical implications (Review). Oncol. Rep..

[76] McCormick R.I., Blick C., Ragoussis J., Schoedel J., Mole D.R., Young A.C., Selby P.J., Banks R.E., Harris A.L. (2013). miR-210 is a target of hypoxia-inducible factors 1 and 2 in renal cancer, regulates ISCU and correlates with good prognosis. Br. J. Cancer.

[77] Schanza L.M., Seles M., Stotz M., Fosselteder J., Hutterer G.C., Pichler M., Stiegelbauer V. (2017). MicroRNAs Associated with Von Hippel-Lindau Pathway in Renal Cell Carcinoma: A Comprehensive Review. Int. J. Mol. Sci..

[78] Shen G., Li X., Jia Y.F., Piazza G.A., Xi Y. (2013). Hypoxia-regulated microRNAs in human cancer. Acta Pharmacol. Sin..

[79] Tang K., Xu H. (2015). Prognostic value of meta-signature miRNAs in renal cell carcinoma: An integrated miRNA expression profiling analysis. Sci. Rep..

[80] Wang Q., Lin W., Tang X., Li S., Guo L., Lin Y., Kwok H.F. (2017). The Roles of microRNAs in Regulating the Expression of PD-1/PD-L1 Immune Checkpoint. Int. J. Mol. Sci..

[81] Yee D., Shah K.M., Coles M.C., Sharp T.V., Lagos D. (2017). MicroRNA-155 induction via TNF-alpha and IFN-gamma suppresses expression of programmed death ligand-1 (PD-L1) in human primary cells. J. Boil. Chem..

[82] Durrani F., Cao S., Park Y.-M., Thiompson G., Martin J., Yang G., Kuettel M., Rustum Y.M. (2007). Synergistic effect of selenium compounds with radiation therapy in human A549 lung xenografts. Cancer Res..

[83] Durrani F.A., Chintala S., Toth K., Cao S., Rustum Y.M. (2015). Mechanism-based drug combination targeting HIF-2α and VEGF in renal cancer xenografts. Trends Cell Mol. Boil..

[84] Frost J., Galdeano C., Soares P., Gadd M.S., Grzes K.M., Ellis L., Epemolu O., Shimamura S., Bantscheff M., Grandi P. (2016). Potent and selective chemical probe of hypoxic signalling downstream of HIF-alpha hydroxylation via VHL inhibition. Nat. Commun..

[85] Soares P., Gadd M.S., Frost J., Galdeano C., Ellis L., Epemolu O., Rocha S., Read K.D., Ciulli A. (2018). Group-Based Optimization of Potent and Cell-Active Inhibitors of the von Hippel-Lindau (VHL) E3 Ubiquitin Ligase: Structure-Activity Relationships Leading to the Chemical Probe (2*S*,4*R*)-1-((*S*)-2-(1-Cyanocyclopropanecarboxamido)-3,3-dimethylbutanoyl)-4-hydroxy-*N*-(4-(4-methylthiazol-5-yl)benzyl)pyrrolidine-2-carboxamide (VH298). J. Med. Chem..

[86] Courtney K.D., Infante J.R., Lam E.T., Figlin R.A., Rini B.I., Brugarolas J., Zojwalla N.J., Lowe A.M., Wang K., Wallace E.M. (2018). Phase I Dose-Escalation Trial of PT2385, a First-in-Class Hypoxia-Inducible Factor-2alpha Antagonist in Patients with Previously Treated Advanced Clear Cell Renal Cell Carcinoma. J. Clin. Oncol..

[87] Kim S., Lee E., Jung J., Lee J.W., Kim H.J., Kim J., Yoo H.J., Lee H.J., Chae S.Y., Jeon S.M. (2018). microRNA-155 positively regulates glucose metabolism via PIK3R1-FOXO3a-cMYC axis in breast cancer. Oncogene.

[88] Gao Y., Ma X., Yao Y., Li H., Fan Y., Zhang Y., Zhao C., Wang L., Ma M., Lei Z. (2016). miR-155 regulates the proliferation and invasion of clear cell renal cell carcinoma cells by targeting E2F2. Oncotarget.

[89] Yao R., Ma Y.L., Liang W., Li H.H., Ma Z.J., Yu X., Liao Y.H. (2012). MicroRNA-155 modulates Treg and Th17 cells differentiation and Th17 cell function by targeting SOCS1. PLoS ONE.

[90] Ivan M., Harris A.L., Martelli F., Kulshreshtha R. (2008). Hypoxia response and microRNAs: No longer two separate worlds. J. Cell. Mol. Med..

[91] Nguyen D.D., Chang S. (2017). Development of Novel Therapeutic Agents by Inhibition of Oncogenic MicroRNAs. Int. J. Mol. Sci..

[92] Schmidt M.F. (2014). Drug target miRNAs: Chances and challenges. Trends Biotechnol..

[93] Wigerup C., Pahlman S., Bexell D. (2016). Therapeutic targeting of hypoxia and hypoxia-inducible factors in cancer. Pharmacol. Ther..

[94] Jedeszko C., Paez-Ribes M., Di Desidero T., Man S., Lee C.R., Xu P., Bjarnason G.A., Bocci G., Kerbel R.S. (2015). Postsurgical adjuvant or metastatic renal cell carcinoma therapy models reveal potent antitumor activity of metronomic oral topotecan with pazopanib. Sci. Transl. Med..

[95] Oevermann K., Buer J., Hoffmann R., Franzke A., Schrader A., Patzelt T., Kirchner H., Atzpodien J. (2000). Capecitabine in the treatment of metastatic renal cell carcinoma. Br. J. Cancer.

[96] Tannir N.M., Thall P.F., Ng C.S., Wang X., Wooten L., Siefker-Radtke A., Mathew P., Pagliaro L., Wood C., Jonasch E. (2008). A phase II trial of gemcitabine plus capecitabine for metastatic renal cell cancer previously treated with immunotherapy and targeted agents. J. Urol..

[97] Cao S., Durrani F.A., Rustum Y.M. (2004). Selective modulation of the therapeutic efficacy of anticancer drugs by selenium containing compounds against human tumor xenografts. Clin. Cancer Res..

[98] Cao S., Durrani F.A., Toth K., Rustum Y.M. (2014). Se-methylselenocysteine offers selective protection against toxicity and potentiates the antitumour activity of anticancer drugs in preclinical animal models. Br. J. Cancer.

[99] Zakharia Y., Garje R., Brown J.A., Nepple K.G., Dahmoush L., Gibson-Corley K. (2018). Phase1 clinical trial of high doses of Seleno-L-methionine (SLM), in sequential combination with axitinib in previously treated and relapsed clear cell renal cell carcinoma (ccRCC) patients. J. Clin. Oncol..

[100] Zakharia Y., Bhattacharya A., Rustum Y.M. (2018). Selenium targets resistance biomarkers enhancing efficacy while reducing toxicity of anti-cancer drugs: Preclinical and clinical development. Oncotarget.

